# HPV vaccination and factors influencing vaccine uptake among people of Indian ancestry living in the United States

**DOI:** 10.1017/S0950268822001315

**Published:** 2022-07-27

**Authors:** Philip Ratnasamy, Anees B. Chagpar

**Affiliations:** Department of Surgery, Yale University School of Medicine, New Haven, CT, USA

**Keywords:** Disparities, human papilloma virus, vaccines

## Abstract

Approximately one-quarter of annual global cervical cancer deaths occur in India, possibly due to cultural norms promoting vaccine hesitancy. We sought to determine whether people of Indian ancestry (POIA) in the USA exhibit disproportionately lower human papilloma virus (HPV) vaccination rates than the rest of the US population. We utilised the 2018 National Health Interview Survey to compare HPV vaccine initiation and completion rates between POIA and the general US population and determined factors correlating with HPV vaccine uptake among POIA. Compared to other racial groups, POIA had a significantly lower rate of HPV vaccination (8.18% *vs.* 12.16%, 14.70%, 16.07% and 12.41%, in White, Black, Other Asian and those of other/mixed ancestry, respectively, *P* = 0.003), but no statistically significant difference in vaccine series completion among those who received at least one injection (3.17% *vs.* 4.27%, 3.51%, 4.31% and 5.04%, *P* = 0.465). Among POIA, younger individuals (*vs.* older), single individuals (*vs.* married), those with high English proficiency (*vs.* low English proficiency), those with health insurance and those born in the USA (*vs.* those born outside the USA) were more likely to obtain HPV vaccination (*P* = 0.018, *P* = 0.006, *P* = 0.029, *P* = 0.020 and *P* = 0.019, respectively). Public health measures promoting HPV vaccination among POIA immigrants may substantially improve vaccination rates among this population.

## Introduction

Cervical cancer is the fourth most common cancer in women worldwide with 570 000 diagnosed cases in 2018 [1]. India accounts for one-quarter of *all* global cervical cancer cases annually while only comprising 16–17% of the world's population [2, 3]. Estimates suggest that 1 in 53 women living in India will develop cervical cancer during their lifetime, sharply contrasting with 1 in 100 women living in developed nations [3].

Currently, human papilloma virus (HPV) vaccination is the premier tool to prevent HPV infection and subsequent development of cervical cancer. Without immunisation, approximately 30 of every 1000 women would develop cervical cancer during their lifetime; however, with vaccination, this drops to 10 of every 1000 women [4]. Bivalent and quadrivalent HPV vaccines were licensed in India in 2008; a nonavalent vaccine became available in 2018 [5]. Despite the availability of vaccines, however, there remains ‘much misinformation, ambiguity, controversy, and confusion surrounding the safety, duration of immunity, validity of endpoints chosen in trials, effectiveness, and cost-effectiveness of HPV vaccines, and moral and cultural issues’ [5] that potentially deter Indian women from taking the vaccine.

Currently, a three-dose quadrivalent HPV vaccination is recommended to girls and boys aged 9–26 years old [6]. Some adults aged 27–45 may also receive the HPV vaccine if deemed appropriate by their physician [6]. When indicated, HPV vaccines are typically administered by primary care physicians during regular doctor's office visits (i.e. wellness visits, examinations, etc.) [7]; however, vaccinations may be obtained through other means as well including paediatricians, OBGyns, preventative health clinics, etc. It remains unclear whether people of Indian ancestry (POIA), who move to the USA, continue to have disproportionately lower HPV vaccination rates than the rest of the US population. We set out to explore this question using a large national population-based dataset, and to further elucidate factors that may affect vaccination rates among POIA in the USA.

## Methods

The National Health Interview Survey (NHIS) is the largest source of health information for Americans. This is a cross-sectional household interview survey conducted in a face-to-face format by the Centers for Disease Control and Census personnel using computer-assisted interviewing techniques. The survey utilises a complex hierarchical sampling scheme that is designed to be representative of the entire civilian non-institutionalised US population. The NHIS collects data on numerous aspects of health, including respondent demographics, health behaviours, healthcare access and utilisation and several others. The data are publicly available and the questionnaires for the survey can be found here (https://ftp.cdc.gov/pub/Health_Statistics/NCHS/Survey_Questionnaires/NHIS/2018/english/qadult.pdf).

For this analysis, we utilised the 2018 NHIS, which surveyed 72 831 individuals. The response rate for sample adults surveyed was 83.9% (61 105 individuals). We compared HPV vaccine initiation and completion rates between POIA and the general US population, and factors correlating with HPV vaccine uptake among POIA. Covariates included age, education level, gender, marital status, proficiency in speaking English, whether an individual was born in the USA, marital status, ratio of family income to the federal poverty level and insurance status. We defined HPV vaccine initiation as having had at least one dose of the HPV vaccine, and completion as having had at least three doses. We recognise that the guidelines for completion of HPV vaccination recently changed in 2016; however, for the majority of the individuals in our cohort (who were aged 18–64 at the time of the survey in 2018), vaccination completion would have been defined by the older definition of at least three doses.

Statistical analyses were performed using SAS-callable SUDAAN Software (RTI International, Research Triangle Park, NC). Wald chi-square analyses were used for bivariate analyses to evaluate variations in HPV vaccine initiation and completion rates by patient demographic factors. Significance in this analysis was per variable. For those variables which were found to be significant on bivariate analysis, a multivariate logistic regression was performed to determine factors independently associated with HPV vaccine uptake among POIA. Odds ratios with 95% confidence levels are provided to demonstrate the relative impact of each level or category within variables on the outcome variable; the overall significance level is provided to demonstrate the impact of each variable on the overall outcome.

As the NHIS is a publicly available de-identified dataset, this study was deemed exempt by the Yale Human Investigations Committee.

## Results

In the 2018 NHIS, 17 004 people between the ages of 18 and 64 responded to questions regarding racial ancestry and HPV vaccination. As the NHIS is designed to be representative of the population, these individuals represented 185 065 802 in the US population. Of this cohort, 1.69% [287] identified themselves as being of Indian ancestry, representing 3 127 612 POIA in the USA. POIA had a significantly lower rate of HPV vaccine initiation than other races (8.18% *vs.* 12.16%, 14.70%, 16.07% and 12.41%, for White, Black, Other Asian and those of other/mixed ancestry, respectively, *P* = 0.003). Of those who received at least one dose of the HPV vaccine, POIA vaccine series completion was not statistically significantly different from other racial groups (3.17% *vs.* 4.27%, 3.51%, 4.31% and 5.04% for POIA *vs.* White, Black, Other Asian and those of other/mixed ancestry, respectively, *P* = 0.465).

Among POIA, we analysed factors that affected initiation of HPV vaccination (i.e. getting at least one dose; Table 1). Not surprisingly, younger women tended to have higher rates of HPV vaccine initiation. Notably, however, those who were born in the USA had a nearly 10-fold higher rate of HPV vaccine initiation than those who immigrated to this country (41.78% *vs.* 4.85%, *P* = 0.0186), although the duration of US residence did not significantly affect vaccine uptake in the POIA population (*P* = 0.5017). While proficiency in the English language was a strong predictor of HPV vaccine uptake among POIA (*P* = 0.0286) and all respondents who got at least one dose of the HPV vaccine were very proficient in English, education level did not affect HPV vaccine initiation rates (*P* = 0.2310). Similarly, while having health insurance was a key factor in receipt of the HPV vaccine (*P* = 0.0204), and all who claimed to have received at least one dose were insured, level of family income did not play role in HPV vaccine initiation among POIA (*P* = 0.9072). On multivariable analysis, being born in the USA and having insurance were independent predictors of HPV vaccine uptake among POIA (Table 2). In contrast to HPV vaccine initiation, there were no socio-demographic factors that significantly affected HPV vaccine completion among POIA who had received at least one dose (Table 3).

**Table 1.** Factors affecting HPV vaccine initiation among POIA

Factor	Vaccine initiation rate (%)	*P*-value
Region of US residence		0.7198
Northeast	8.17	
Midwest	6.13	
South	11.14	
West	5.04	
Age		0.018
18–25	38.12	
26–45	6.45	
46–64	1.79	
Education level		0.2310
<HS Diploma	0.00	
HS Diploma	5.61	
Some college	17.69	
Bachelors	3.17	
Masters	5.43	
Prof/Doctorate	38.36	
Sex		0.9490
Male	8.04	
Female	8.32	
Born in the USA		0.0186
Yes	41.78	
No	4.85	
Years living in the USA		0.5017
<1 year	22.11	
1–5 years	9.18	
5–10 years	3.37	
10–15 years	9.35	
15+ years	2.31	
English proficiency		0.0286
Very well	8.75	
Not well	0.00	
Not at all	0.00	
Having insurance		0.0204
Have insurance	8.68	
No insurance	0.00	
Family income (relative to FPL)		0.9072
<1.99×	8.57	
2×–4×	8.08	
Marital status		0.0058
Married/partnered	3.11	
Single	29.53	
Widowed, separated or divorced	0	

FPL, federal poverty level.

**Table 2.** Multivariable analysis of factors affecting HPV vaccine uptake among POIA in the USA

Factor	OR (95% CI)	*P*-value
Age (*vs.* >45)		0.1764
<26	20.03 (2.50–160.61)	
26–45	2.25 (0.38–13.41)	
Born in the USA (*vs.* not)	7.20 (2.53–20.54)	0.0002
Have insurance (*vs.* not)	6335.21 (2575.79–15 581.56)	<0.0001
Speak English very well (*vs.* not at all)	1.00 (1.00–1.00)	1.000
Single (*vs.* Married/partnered)	4.29 (1.02–17.95)	0.0857

OR, odds ratio; 95% CI, 95% confidence interval.

**Table 3.** Factors affecting HPV vaccination completion among POIA who had received at least one dose of the HPV vaccine

Factor	Vaccine completion rate (%)	*P*-value
Region of US residence		0.6012
Northeast	4.19	
Midwest	1.49	
South	4.57	
West	1.22	
Age		0.1282
18–25	17.97	
26–45	2.78	
46–64	0.39	
Education level		0.5554
<HS Diploma	0.00	
HS Diploma	1.13	
Some college	9.60	
Bachelors	1.15	
Masters	1.08	
Prof/Doctorate	13.55	
Sex		0.9882
Male	3.15	
Female	3.19	
Born in the USA		0.0819
Yes	27.05	
No	1.36	
Years living in the USA		0.4235
<1 year	22.11	
1–5 years	0.76	
5–10 years	0.00	
10–15 years	0.88	
15+ years	1.67	
English proficiency		0.1044
Very well	3.43	
Not well	0.00	
Not at all	0.00	
Having insurance		0.0619
Have insurance	3.34	
No insurance	0.00	
Ratio of income to federal poverty level		0.2751
<1.99×	5.90	
2×–4×	2.41	
Marital status		0.0954
Married/partnered	0.46	
Single	16.98	
Widowed, separated or divorced	0	

## Discussion

In the USA, HPV vaccination rates remain much lower than recommended by public health officials, leading to thousands of preventable instances of cervical cancer each year [8, 9]. Disparities in the incidence of cervical cancer between Blacks, Hispanics and Whites living in the USA are thoroughly discussed in the literature. In contrast – likely in part due to the ‘model minority’ stereotype – relatively few studies have explored HPV vaccine uptake and cervical cancer diagnosis among Asian Americans, with even fewer studying people of south-Asian decent (i.e. POIA) [10–12]. Through our analysis of NHIS data, we found that POIA between the ages of 18 and 64 exhibit significantly lower HPV vaccine initiation rates than members of other racial groups living in the USA. This discovery aligns with other findings regarding HPV vaccination among Asian Americans in general. For instance, one study examined the 2013–2016 National Health and Nutrition Examination Surveys (NHANES) and found that Asians between the ages of 9 and 26 have the lowest HPV vaccination rate of all racial/ethnic groups in the USA [13] 39.6% *vs.* 48.4%, 49.2%, 42.4% and 42.6% for White, Black, Mexican American and ‘Other Hispanic’ Americans, respectively [13]. Commonly reported barriers to HPV vaccination among Asian Americans include: lack of provider recommendation [14–16], belief that only promiscuous women are at risk of HPV infection and cervical cancer [17], lack of HPV vaccine education by healthcare providers [18] and cultural factors causing sexual health stigma and hesitancy towards utilising women's health services [17].

While the NHIS did not specifically field questions as to why POIA had a lower vaccination rate than others in the US population, our analysis evaluating differences between those who got an HPV vaccine *vs.* those who did not shed some light on factors that predict vaccine uptake in this population. For example, we found that POIA who were born in the USA had a significantly higher rate of initiating HPV vaccination than those who immigrated. This seemed to be independent of English language proficiency or level of education and speaks potentially to the effect of acculturation and/or access on vaccine uptake. Other studies have similarly found that individuals born in the USA initiated the HPV vaccine series at twice the rate of those born outside the USA [19]. Indeed, some have suggested that POIA born in the USA may have greater access to HPV education and vaccination than those born outside the USA and may face less stigma in discussing or seeking treatment for sexual health-related matters [18, 20]. While it would have been nice to look at age of immigration (and whether this was before or after the recommended age for the HPV vaccine), the NHIS is limited in that it does not contain this information. This, however, would be interesting to evaluate in further studies.

In our analysis, all patients who obtained an HPV vaccine stated they spoke English very well. This correlation of HPV vaccination with high levels of English proficiency is consistent with other studies which have found that Asian Americans commonly cite language barriers as a reason underlying their lack of HPV vaccination [17], and those with higher English fluency are more receptive to receiving HPV vaccination for themselves and their families [21, 22]. Again, age at immigration may be correlated with English language proficiency. Unfortunately, the NHIS does not provide data on age at immigration or duration of residence. This would be interesting to evaluate in further studies.

In addition, while one may have anticipated a correlation between HPV vaccine uptake and educational status, we did not find this. Taylor *et al*. similarly found no correlation between educational status and HPV vaccination uptake in their study of Cambodian Americans [15]. This may be related to the fact that there may be a decoupling of socio-cultural beliefs and academic education in terms of whether Asians, and particularly POIA, opt to take the HPV vaccine.

Also surprisingly, we found no relationship between family income relative to the federal poverty level and POIA HPV vaccine uptake; however, data regarding the relationship between socio-economic status and vaccine uptake in the USA have been mixed [23]. For instance, one study found low income to be a barrier to vaccination [24], while another study found high-income as a barrier to vaccination [25]. Thus it seems that perceived cost, rather than simply income level, is a key barrier to obtaining the HPV vaccine among Asian Americans [26–28].

Indeed we, like many studies evaluating a plethora of health maintenance and disease prevention strategies, found that insurance coverage to be a critical factor for uptake of HPV vaccine among POIA. Hopfer *et al*. in their narrative analysis, found restrictive insurance policies to be a significant barrier to HPV vaccine uptake among Asian Americans [29], such that some Asian Americans forgo receiving HPV vaccination due to uncertainty regarding whether their insurance policy covers the vaccine [14]. Our finding on the impact of insurance status on HPV vaccine uptake among POIA was independent of family income, education, place of birth or English proficiency, suggesting that provision of HPV vaccines as part of a federally provided mandate (regardless of insurance status) may improve vaccination rates.

Our finding that younger women were more likely to have received the HPV vaccine was not unexpected. Other studies have similarly found that younger Asian American individuals are more likely to initiate HPV vaccination [30]. When the HPV vaccine initially became available in the USA in 2006, it was initially recommended only for cervical cancer prevention, with girls between the ages of 9 and 26 being targeted for vaccination. More recently in 2019, guidance has expanded to include a strategy of ‘shared decision making’ for HPV vaccination for all adults (regardless of gender) through age 45. This, however, occurred after the 2018 NHIS survey, upon which the current study is based. Interestingly, some studies found that parental belief that their children were *too* young to obtain HPV vaccination decreased uptake in Asian American children [14]. Given that, as of 2018, HPV vaccination was received in a doctor's office setting by 79.2% of recipients aged 13–17 in the USA, parental beliefs could strongly influence HPV vaccine uptake among POIA youth [7].

While we, like others, found that single individuals are more likely to obtain HPV vaccination than married people [31, 32], this may be related to the fact that single people are more likely to be younger, and therefore this variable may be collinear with age. Indeed, on our multivariable analysis, neither age nor marital status were independent predictors of HPV vaccination. Some, however, have argued that married individuals perceive their risk of HPV infection as low due to having a single sexual partner, whereas those who are single may perceive themselves as being at higher risk of HPV infection due to having multiple sexual partners [33].

Regarding HPV vaccine series completion rates, we found that POIA between the ages of 18 and 64 who initiate HPV vaccination are equally likely to complete the three-dose series compared to other major racial groups in the USA. These data echo findings from the 2018 National Immunization Teen Survey. This work found that Asian American teenagers (aged 13–17) completed the HPV vaccine series at roughly the same rate as White, Black and Hispanic teenagers (53.1% *vs.* 47.8%, 53.3% and 56.3%, respectively) [34]. Interestingly, Agénor *et al*., evaluating data from the 2015 NHIS, found that Asian women had significantly lower odds of completing the HPV vaccination series when compared to White women [35]. While they did not subclassify Asian women as we did, the 2018 NHIS data do not seem to suggest a significant difference in HPV completion rates among women who initiated HPV vaccination by the racial group (*P* = 0.7658), although a difference is seen in initiation rates (*P* = 0.0091), echoing our findings among POIA. These data suggest that racial disparities in HPV vaccination rates that may have existed in the past may have dissipated in recent years.

Our data should be viewed in light of its inherent limitations. As a survey study, it is limited by the questions fielded and is subject to recall bias. Furthermore, we recognise that in 2016, guidelines changed to allow for two doses of HPV instead of three, and our study defined completion of the series using the older definition which would have applied to the majority of people in our cohort. We recognise, however, there was some variability in the guidelines and there was no way for our study to control for this.

Despite these limitations, our study has a number of strengths. Given the design of the NHIS which is representative of the entire US population, this dataset gives us key insights into factors affecting HPV vaccination rates among POIA. Importantly, while our data demonstrate that POIA are less likely to receive an HPV vaccine than other racial groups, they are just as likely to complete their vaccination schedule once started. As such, targeted public health measures increasing awareness and acceptance of HPV vaccination among POIA may be particularly effective in increasing immunity to HPV among this community. Furthermore, our finding that those born in the USA were more likely to get vaccinated provides hope that, with acculturation and growing acceptance of the vaccine among those born here, HPV vaccination will be higher among POIA in the USA than it is in their ancestral homeland. Among those POIA who continue to migrate to the USA, recognition of disparities in HPV vaccine uptake by clinicians is crucial to increasing HPV vaccination rates within this population. Given recent policy changes allowing for HPV vaccine administration in both men and women up to 45 years old, clinicians should enquire about and discuss HPV vaccination with their migrant POIA patients to promote shared decision making and increased HPV vaccine uptake in this population. Finally, our data highlight insurance as being a key factor that can enhance HPV vaccination rates, suggesting that making HPV vaccines available regardless of insurance status may be a key public health measure to improve vaccination rates and ultimately reduce preventable malignancies associated with HPV – not just for POIA, but for the population as a whole.

## Data Availability

The data analysed during the current study are available in the 2018 National Health Interview Survey at https://www.cdc.gov/nchs/nhis/1997-2018.htm.
